# Supplementary material for: Variation in bradyrhizobial NopP effector determines symbiotic incompatibility with Rj2-soybeans via effector-triggered immunity
Source: Nat Commun. 2018 Aug 7;9:3139. doi: 10.1038/s41467-018-05663-x (PMC6081438; doi:10.1038/s41467-018-05663-x)
Supplement: Supplementary file 1 — Supplementary Information [file 41467_2018_5663_MOESM1_ESM.docx]

**Variation in bradyrhizobial NopP effector determines symbiotic incompatibility with *Rj2-*soybeans *via* effector-triggered immunity**

Sugawara *et al*.

**Supplementary Figure 1. Validation of *nopP* mutations in spontaneous mutants.** (**a**) Genetic organization of *nopP* and its flanking region in the genome of *Bradyrhizobium diazoefficiens* USDA 122. Genes are indicated with pentagons: black, *nopP*; gray, *nif* genes. Black arrows indicate the sites corresponding to the primers used for PCR shown in panel **b**. (**b**) Amplification products of the *nopP* region obtained from genomic DNA by PCR with the oligonucleotide primers 09010_F and 09010_R (Supplementary Data 2), and analyzed by agarose gel electrophoresis. The PCR products from the W3-1a and W9-1a mutants were longer than that from wild-type USDA 122. (**c**, **d**) Partial nucleotide sequences of the PCR products shown in panel **b**. ISRj2 and ISRj1 are inserted in the *nopP* coding region in W3-1a and W9-1a, respectively.

**
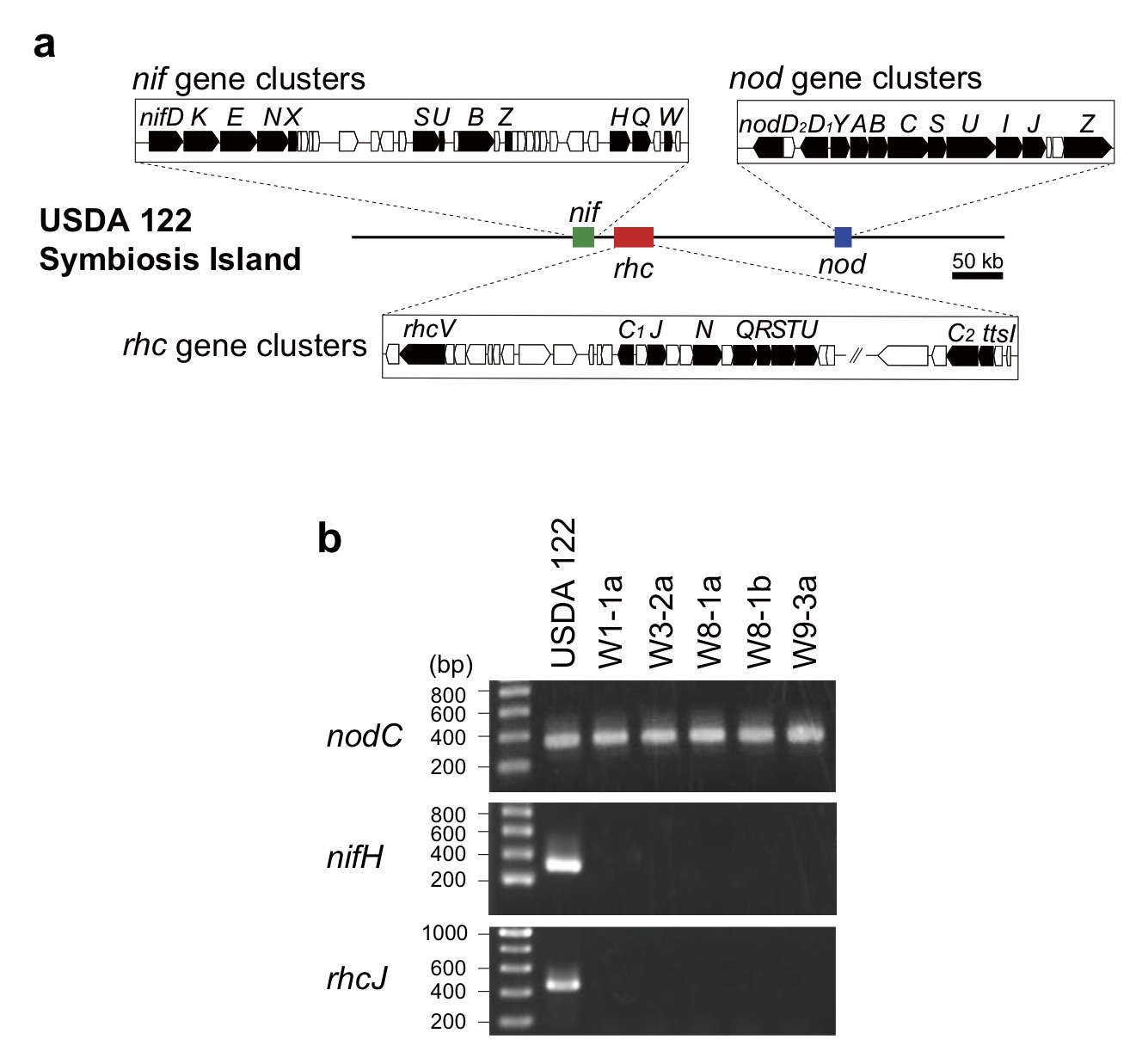
**

**Supplementary Figure 2. Absence of genes involved in nitrogen fixation (*nif*) and type III secretion system (*rhc*) in USDA 122 Nod^+^/Fix^−^ spontaneous mutants.** (**a**) Genetic organization of the *nod*, *nif*, and *rhc* genes in the symbiosis island of *Bradyrhizobium diazoefficiens* USDA 122. (**b**) Detection of the *nodC*, *nifH,* and *rhcJ* genes in the genomic DNA of USDA 122 and its spontaneous mutants by PCR analysis. *nifH* and *rhcJ* genes were not detected in the Nod^+^/Fix**^−^** mutants (W1-1a, W3-2a, W8-1a, W8-1b, and W9-3a). The primers used for each gene are listed in Supplementary Data 2.


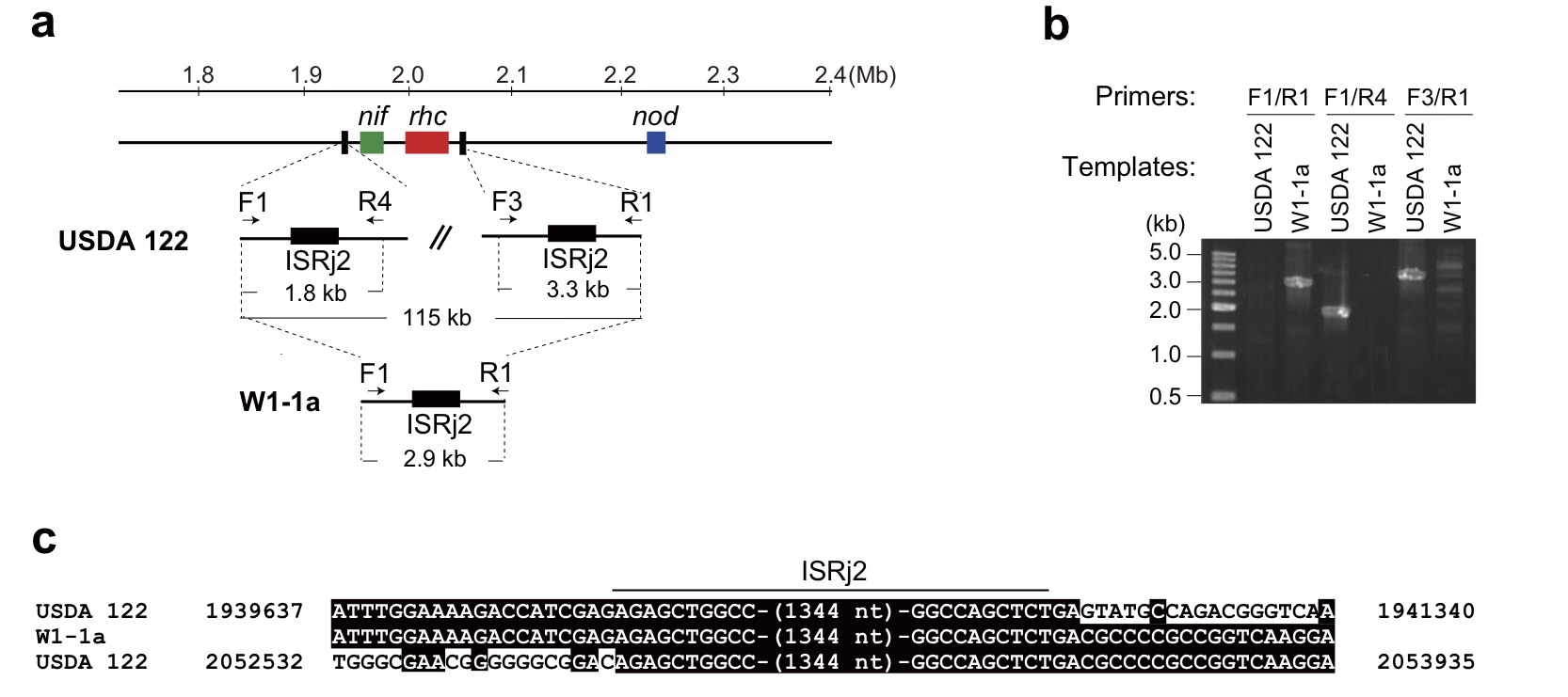


**Supplementary Figure 3. Verification of an insertion sequence element–mediated large deletion in the spontaneous mutant W1-1a.** (**a**) Schematic illustration of the insertion sequence element ISRj2-mediated large deletion at the *nif* and *rhc* loci in the genome of W1-1a. The scale at the top indicates nucleotide positions in the USDA 122 genome (accession no. CP013127). Colored boxes indicate gene cluster regions. Arrows indicate sites corresponding to the primers used for PCR analysis. (**b**) Genomic PCR analysis. The sequences of the oligonucleotide primers Fix-del_F1 (F1), Fix-del_F3 (F3), Fix-del_R1 (R1), and Fix-del_R4 (R4) are listed in Supplementary Data 2. The amplification products were analyzed by agarose gel electrophoresis. (**c**) Nucleotide sequence of the PCR product obtained with the F1 and R1 primers from W1-1a genomic DNA aligned with the corresponding genomic regions of wild-type USDA 122. Numbers indicate nucleotide positions in the USDA 122 genome. The ISRj2-mediated deletion of a large genomic region detected in W1-1a by PCR was confirmed by DNA sequencing.


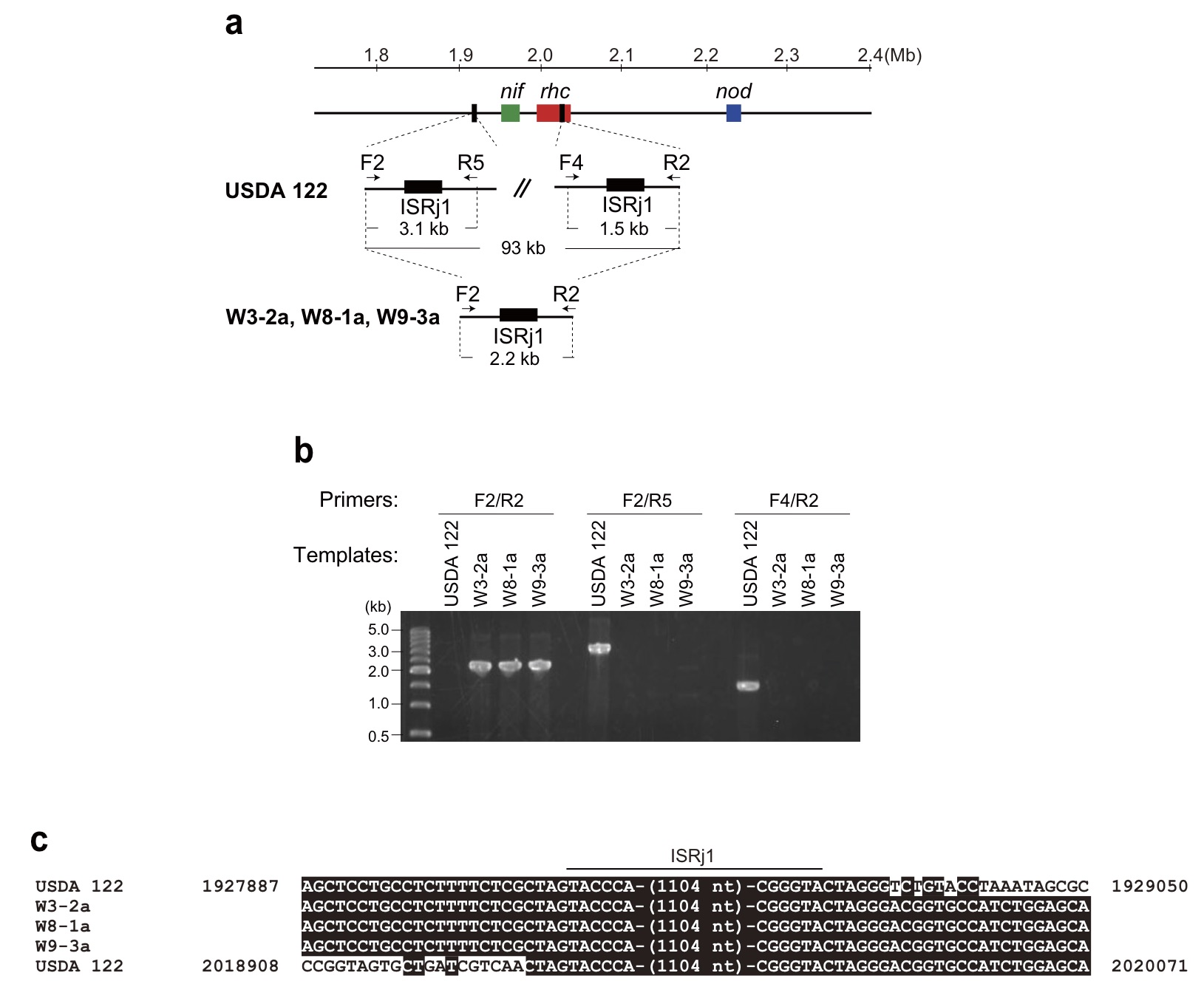


**Supplementary Figure 4. Verification of insertion sequence element–mediated large deletions in the spontaneous mutants W3-2a, W8-1a, and W9-3a.** (**a**) Schematic illustration of the insertion sequence element ISRj1–mediated large deletion at the *nif* and *rhc* loci in the genomes of W3-2a, W8-1a, and W9-3a. The scale at the top indicates nucleotide positions in the USDA 122 genome (accession no. CP013127). Colored boxes indicate gene cluster regions. Arrows indicate sites corresponding to the primers used for PCR analysis. (**b**) Genomic PCR analysis. The sequences of the oligonucleotide primers Fix-del_F2 (F2), Fix-del_F4 (F4), Fix-del_R2 (R2), and Fix-del_R5 (R5) are listed in Supplementary Data 2. The amplification products were analyzed by agarose gel electrophoresis. (**c**) Nucleotide sequences of the PCR products obtained with the F2 and R2 primers from mutant genomic DNAs aligned with the corresponding genomic regions of wild-type USDA 122. Numbers indicate nucleotide positions in the USDA 122 genome. The ISRj1-mediated deletions detected in W3-2a, W8-1a, and W9-3a were confirmed by DNA sequencing.


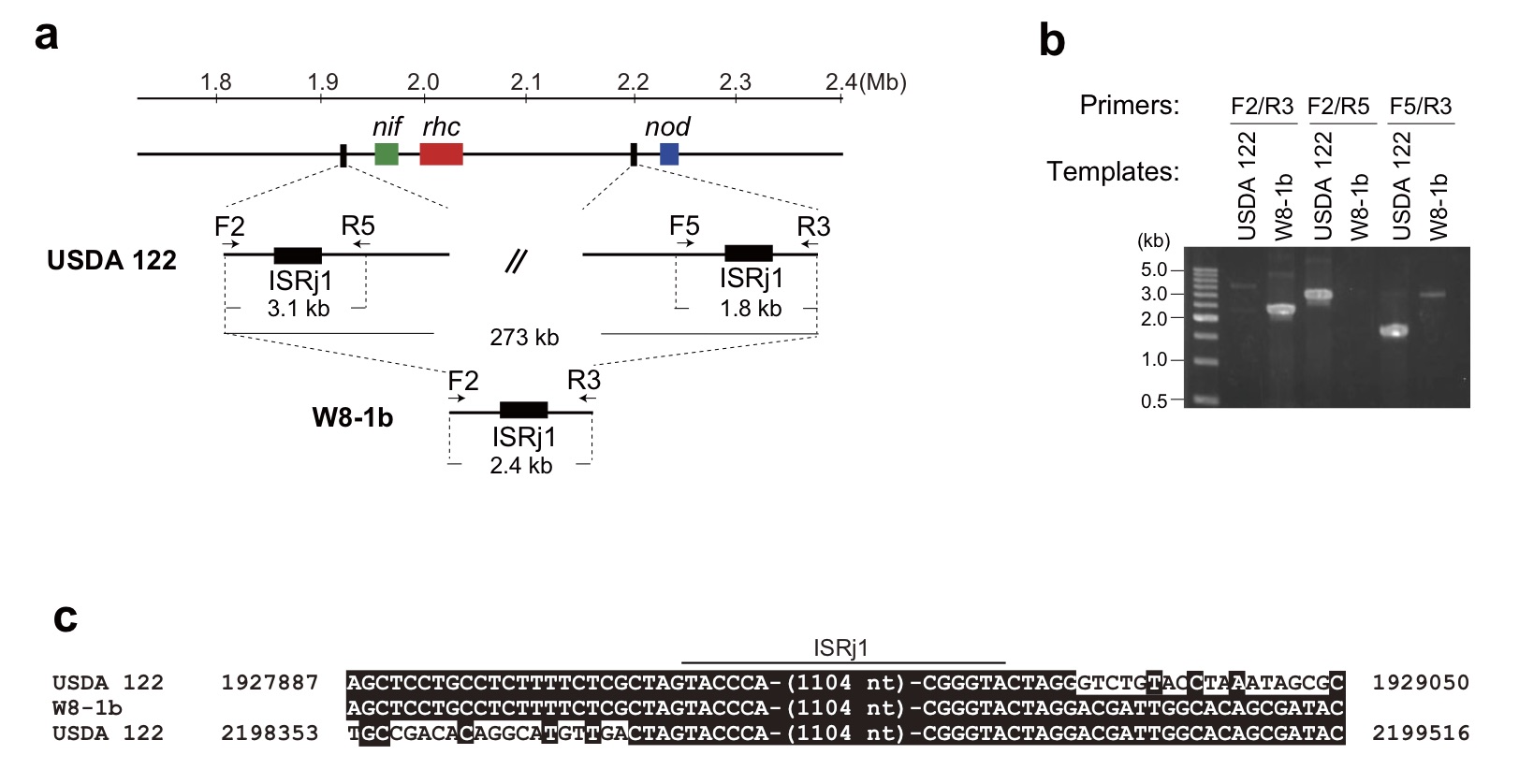


**Supplementary Figure 5. Verification of an insertion sequence element–mediated large deletion in the spontaneous mutant W8-1b.** (**a**) Schematic illustration of an ISRj1-mediated large deletion at the *nif* and *rhc* loci in the genome of W8-1b. The scale at the top indicates nucleotide positions in the USDA 122 genome (accession no. CP013127). Colored boxes indicate gene cluster regions Arrows indicate sites corresponding to the primers used for PCR analysis. (**b**) Genomic PCR analysis. The sequences of the oligonucleotide primers Fix-del_F2 (F2), Fix-del_F5 (F5), Fix-del_R3 (R3), and Fix-del_R5 (R5) are listed in Supplementary Data 2. The amplification products were analyzed by agarose gel electrophoresis. (**c**) Nucleotide sequences of the PCR products obtained with the F3 and R3 primers from W8-1b genomic DNA aligned with the corresponding genomic regions of wild-type USDA 122. Numbers indicate nucleotide positions in the USDA 122 genome. The ISRj1-mediated deletion detected in W8-1b by PCR was confirmed by DNA sequencing.


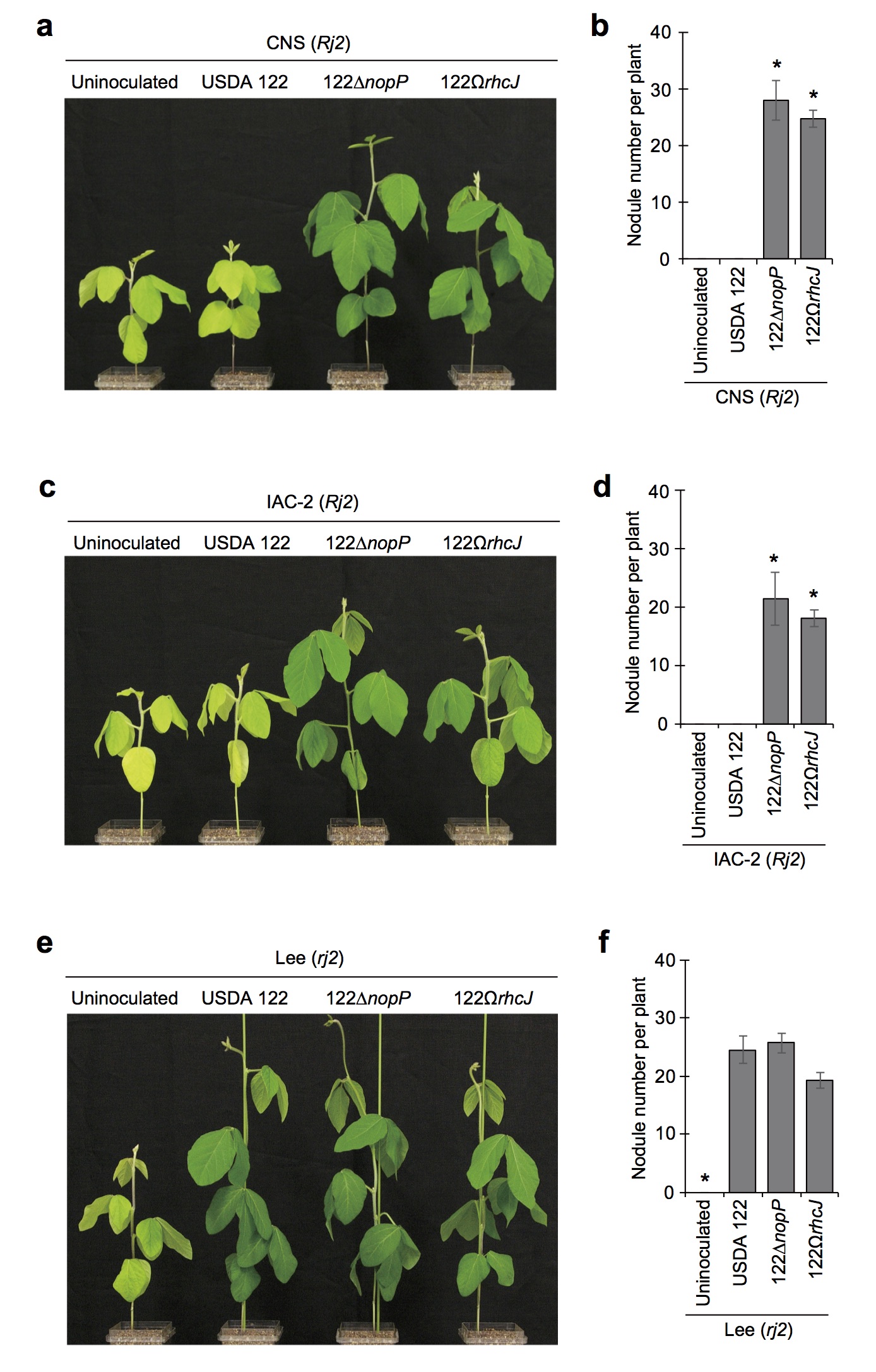


**Supplementary Figure 6. Symbiotic phenotypes of *Rj2* and *rj2* soybean cultivars inoculated with *Bradyrhizobium diazoefficiens* USDA 122, 122∆*nopP*, and 122Ω*rhcJ*.** (**a**, **c**, **e**) Plants at 28 days after inoculation*.* (**b**, **d**, **f**) Numbers of nodules formed on the roots. No experimental repeat was conducted with the same comparison. Error bars show s.e.m. (*n* = 3 or 4). Asterisks above the bars indicate significant difference from USDA 122 (*P* < 0.01, Dunnett’s multiple comparison test).


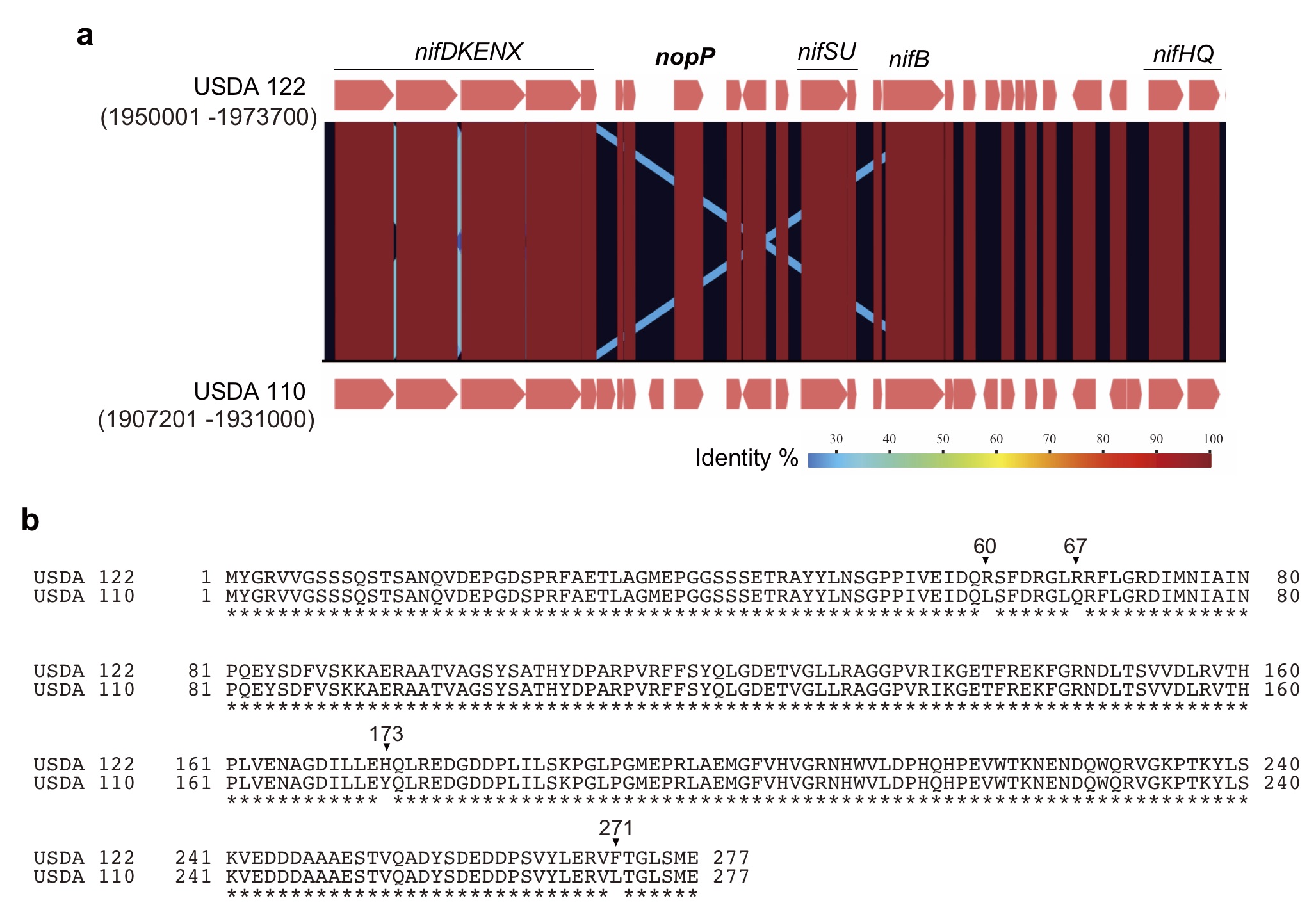


**Supplementary Figure 7. Comparison of *nopP* between *Bradyrhizobium diazoefficiens* USDA 122 and 110^T^.** (**a**) Percentage identity plot of a linear pairwise comparison of the genomic regions including *nopP*. The coding regions were compared by the BLASTP program in GenomeMatcher software​^1^. Numbers in parentheses indicate the ranges of genomic positions used in this comparison. Colors indicate the percentage of amino acid identity. (**b**) Alignment of NopP amino acid sequences from USDA 122 and 110^T^. Asterisks indicate conserved amino acid residues. Arrows and numbers above the sequences indicate the positions of amino acid residues that differ between the strains.


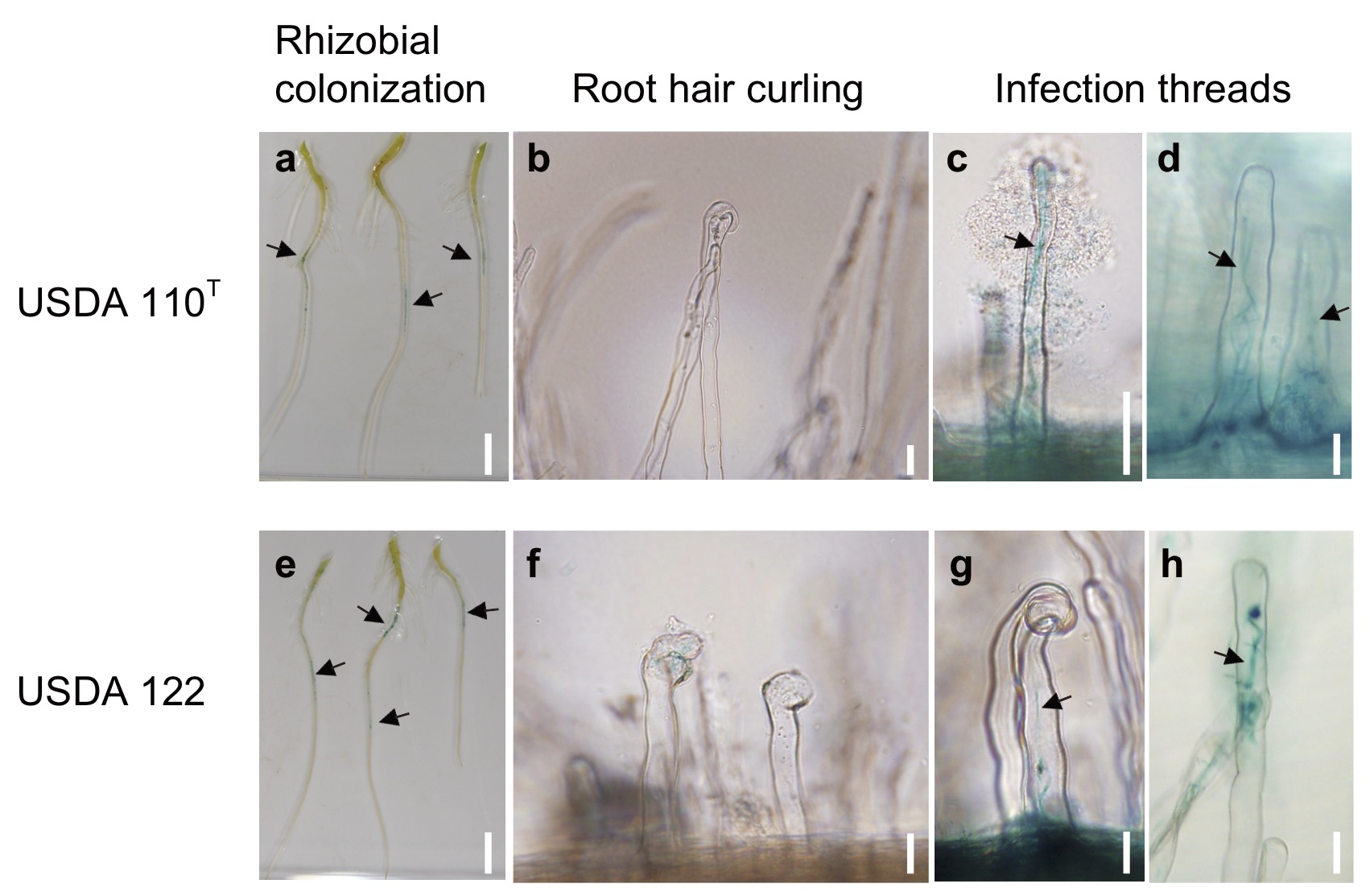


**Supplementary Figure 8. Rhizobial colonization, root-hair curling and infection threads formation in the roots of Hardee (*Rj2*) inoculated with *Bradyrhizobium diazoefficiens* strains.** *gusA*-tagged *B. diazoefficiens* USDA 110^T^ (110*gus*) or USDA 122 (122*gus*) was inoculated onto the seedlings, and images of root and root hair were taken at 2 days after inoculation. All plants shown in the picture were stained with X-Gluc before observation. (**a**) Roots inoculated with USDA 110^T^. (**b, c, d**) Root hairs of Hardee inoculated with USDA 110^T^. (**e**) Roots inoculated with USDA 122. (**f, g, h**) Root hairs of Hardee inoculated with USDA 122. Arrows in panel a and e show blue coloration by X-Gluc staining, indicating rhizobia are colonized. Arrows in the panel c, d, g, h indicate an infection threads. Rhizobial colonization (a, e), root hair curling (b, f) and infection threads formation (c, d, g, h) were observed by inoculation with in both strains. Scale bars, 1 cm (a, e), 20 µm (b, c, d, f, g, h).

**Supplementary Figure 9. Expression of defense marker genes *PR-1* and *PR-5* in roots of Hardee inoculated with *Bradyrhizobium diazoefficiens* USDA 122 and 122*nopP*_110_.** Expression of *PR-1*, *PR-5* in roots of *Glycine max* cv. Hardee was determined by quantitative reverse-transcription PCR using primers listed in Supplementary Data 2 as described in Methods. RNA was isolated from the roots of Hardee at 2 and 4 days after inoculation (DAI). The expression levels of each gene were normalized to each of the *SUBI2* gene. Error bars show s.e.m. of three independent experiments with two plants (from one seed pack). There is no significant difference between all treatments by two-tailed Student’s *t*-test.

**Supplementary Figure 10. Nodulation phenotypes of *B. diazoefficiens* USDA 110 or USDA 122 carrying *nopP* variants on cv. Hardee (*Rj2*).** Number of nodules formed on roots at 28 days after inoculation. Strains and amino acid residues in NopP at positions 60, 67, 173, and 271 are shown on the left, and the nodule numbers are shown on the right. (**a**) *B. diazoefficiens* USDA 110 carrying *nopP* variants were inoculated on Hardee. No experimental repeat was conducted with the same comparison. Error bars show s.e.m. (*n* = 10). (**b**) *B. diazoefficiens* USDA 122, 122*nopP*110, and 122*nopP*_m3 were inoculated on Hardee. Error bars show s.e.m. (*n* = 3). **P* < 0.05, ***P* < 0.01, by two-tailed Student’s *t*-test.

**Supplementary Figure 11. Nodulation phenotypes of *Bradyrhizobium* strains inoculated onto Hardee (*Rj2*).** Nodules were counted at 28 days after inoculation. NopP type was assigned on the basis of the pattern of deduced amino acid sequence (see Supplementary Table 3). No experimental repeat was conducted with the same comparison. Error bars show s.e.m. (*n* = 3). BD, *Bradyrhizobium diazoefficiens*; BJ, *Bradyrhizobium japonicum.*

**
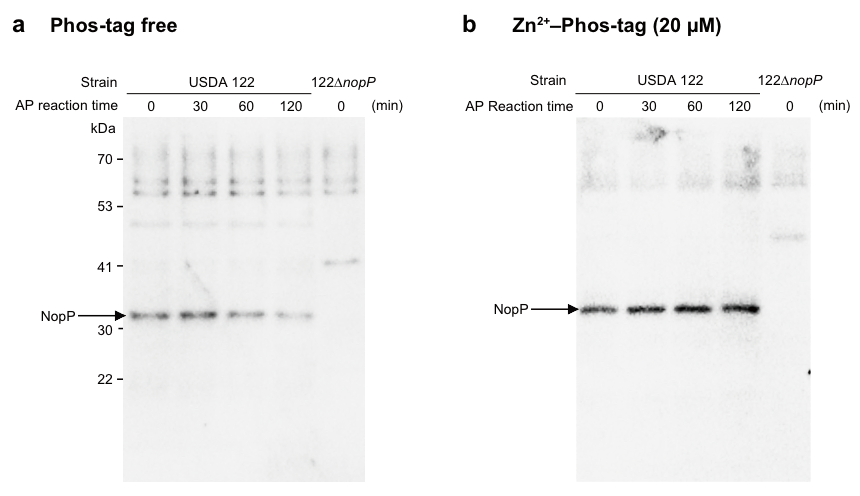
**

**Supplementary Figure 12. Detection of phosphorylated NopP in extracellular proteins of *Bradyrhizobium diazoefficiens* USDA 122.** The extracellular proteins were treated with alkaline phosphatase (AP) and separated by Phos-tag free SDS-PAGE (**a**) or Zn^2+^-Phos-tag SDS-PAGE^12^ (**b**), and immunoreactive bands were detected by Western blotting using anti-NopP. These are representative of two independent experiments. No band shift was detected by AP treatment, suggesting that phosphorylated NopP was not detected using the analysis.


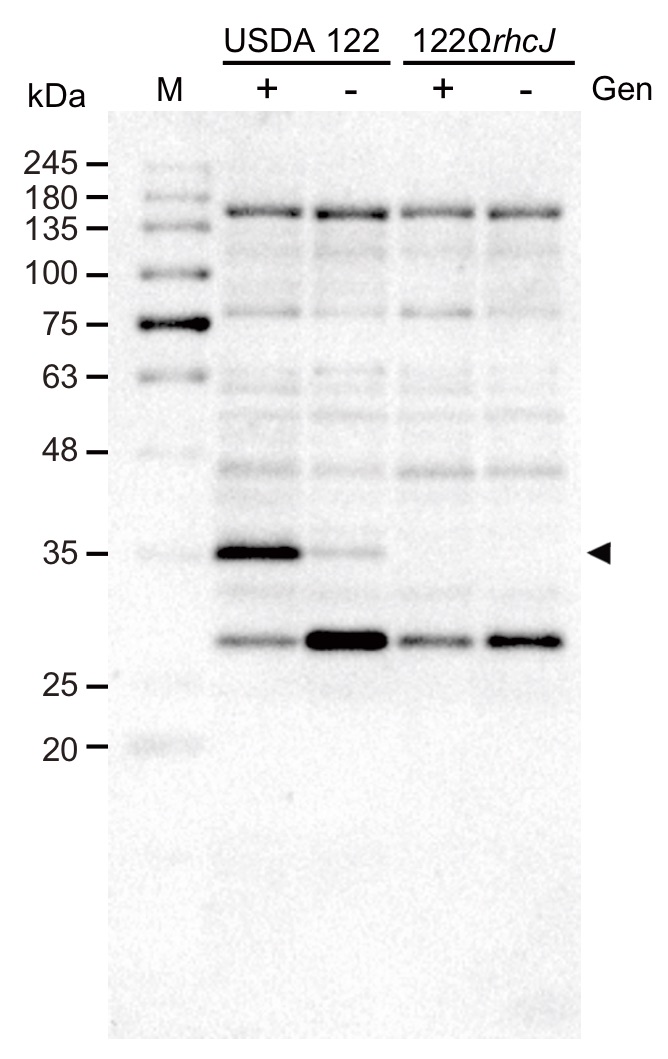


**Supplementary Figure 13. Western blotting analysis of NopP secreted into culture supernatants from *Bradyrhizobium diazoefficiens* USDA 122.** Western blotting analysis of NopP secreted into culture supernatants from USDA 122 and its mutant with inactivated T3SS (122Ω*rhcJ*). USDA 122 or 122Ω*rhcJ* cells were grown in the presence (+) or absence (–) of 10 μM genistein (Gen). The position of NopP is indicated by arrow. M, Protein molecular size marker (BLUeye Prestained Protein Ladder, GeneDireX Inc.) was loaded.

| Strain | Locus tag | Gene | Product and description | Nucleotide change | Amino acid change |
| --- | --- | --- | --- | --- | --- |
| W3-1a | BD122_09010 | *nopP* | Effector protein NopP | c.106insISRj2 | – |
|  | BD122_16130 |  | Histidine kinase | c.1451delC | p.P491HfsX82 |
|  | BD122_19145 |  | Hypothetical protein | c.7723C>A | p.P2575T |
|  |  |  | – | g. 7276157insISBdi2 | – |
|  | BD122_35055 |  | Hypothetical protein | c.103G>A | p.V35M |
|  |  |  | – | g. 8731190_8731191insG | – |
| W9-1a | BD122_09010 | *nopP* | Effector protein NopP | c.593insISRj1 | – |
| W9-1b | BD122_09230 |  | Hypothetical protein, gene adjacent to *rhcV* | c.352G>A, c.352_353insC | p.R118VfsX95 |
|  | BD122_42025 |  | Hypothetical protein | c.259C>A | p.L87I |

**Supplementary Table 1**. Mutations detected in the genomes of spontaneous mutants of *Bradyrhizobium diazoefficiens* USDA 122 with suppressed *Rj2* symbiotic incompatibility.

**Supplementary Table 2**. Strains used in this study to investigate *nopP* diversity.

| Strain | Species | Accession number | | NopP type *^a^* |
| --- | --- | --- | --- | --- |
|  |  | 16S–23S ITS | *nopP* |  |
| USDA 110^T^ | *B. diazoefficiens* | BA000040 | BA000040 | 110 |
| CCBAU 41267 | *B. diazoefficiens* | AJQI01000053 | AJQI01000466 | 110 |
| KS2-1 | *B. diazoefficiens* | AB983955 | [LC331619](https://www.ncbi.nlm.nih.gov/nuccore/LC331619) | 110 |
| YM2-6 | *B. diazoefficiens* | AB983975 | [LC331620](https://www.ncbi.nlm.nih.gov/nuccore/LC331620) | 110 |
| CCBAU 43298 | *B. japonicum* | AJQE01000178 | AJQE01000393 | 110 |
| Is-34 | *B. japonicum* | JRPN01000007 | JRPN01000070 | 110 |
| SEMIA 5079 | *B. japonicum* | CP007569 | CP007569 | 110 |
| FK1-5 | *B. japonicum* | AB984026 | [LC331621](https://www.ncbi.nlm.nih.gov/nuccore/LC331621) | 110 |
| TS5-6 | *B. japonicum* | AB984022 | [LC331622](https://www.ncbi.nlm.nih.gov/nuccore/LC331622) | 110 |
| USDA 122 | *B. diazoefficiens* | CP013127 | CP013127 | 122 |
| Is-1 | *B. diazoefficiens* | LGUJ01000015 | LGUJ01000050 | 122 |
| SEMIA 5080 | *B. diazoefficiens* | ADOU02000007 | ADOU02000002 | 122 |
| KM3-4 | *B. diazoefficiens* | AB984061 | [LC331623](https://www.ncbi.nlm.nih.gov/nuccore/LC331623) | 122 |
| J5 | *B. japonicum* | CP017637 | CP017637 | 122 |
| CCBAU 15618 | *B. japonicum* | AJPZ01000581 | AJPZ01000493 | 122 |
| HK7-6 | *B. japonicum* | AB983913 | [LC331624](https://www.ncbi.nlm.nih.gov/nuccore/LC331624) | 122 |
| TS4-11 | *B. japonicum* | AB984016 | [LC331625](https://www.ncbi.nlm.nih.gov/nuccore/LC331625) | 122 |
| CCBAU 15635 | *B. japonicum* | AJQH01000168 | AJQH01000357 | ST1 |
| KW1-63 | *B. japonicum* | AB983938 | [LC331626](https://www.ncbi.nlm.nih.gov/nuccore/LC331626) | ST1 |
| USDA 4 | *B. japonicum* | AB100740 | AXAF01000044 | ST2 |
| USDA 6^T^ | *B. japonicum* | AP012206 | AP012206 | ST2 |
| USDA 38 | *B. japonicum* | AB100743 | AXAG01000045 | ST2 |
| E109 | *B. japonicum* | CP010313 | CP010313 | ST2 |
| FN1 | *B. japonicum* | JGCL01000002 | JGCL01000038 | ST2 |
| CCBAU 15615 | *B. japonicum* | AJQG01000178 | AJQG01000331 | ST2 |
| CCBAU 15544 | *B. japonicum* | AJQF01000204 | AJQF01000506 | ST2 |
| CCBAU 25435 | *B. japonicum* | AJQA01000448 | AJQA01000397 | ST2 |
| HK1-1 | *B. japonicum* | AB983863 | [LC331627](https://www.ncbi.nlm.nih.gov/nuccore/LC331627) | ST2 |
| HK4-1 | *B. japonicum* | AB983889 | [LC331628](https://www.ncbi.nlm.nih.gov/nuccore/LC331628) | ST2 |
| HK4-8 | *B. japonicum* | AB983891 | [LC331629](https://www.ncbi.nlm.nih.gov/nuccore/LC331629) | ST2 |
| HK4-11 | *B. japonicum* | AB983893 | [LC331630](https://www.ncbi.nlm.nih.gov/nuccore/LC331630) | ST2 |
| HK6-1 | *B. japonicum* | AB983906 | [LC331631](https://www.ncbi.nlm.nih.gov/nuccore/LC331631) | ST2 |
| KM3-9 | *B. japonicum* | AB984066 | [LC331632](https://www.ncbi.nlm.nih.gov/nuccore/LC331632) | ST2 |
| KW1-4 | *B. japonicum* | AB983933 | [LC331633](https://www.ncbi.nlm.nih.gov/nuccore/LC331633) | ST2 |
| KW1-6 | *B. japonicum* | AB983934 | [LC331634](https://www.ncbi.nlm.nih.gov/nuccore/LC331634) | ST2 |
| MY2-3 | *B. japonicum* | AB984085 | [LC331635](https://www.ncbi.nlm.nih.gov/nuccore/LC331635) | ST2 |
| MY2-6 | *B. japonicum* | AB984088 | [LC331636](https://www.ncbi.nlm.nih.gov/nuccore/LC331636) | ST2 |
| TS4-21 | *B. japonicum* | AB984018 | [LC331637](https://www.ncbi.nlm.nih.gov/nuccore/LC331637) | ST2 |
| KG1-2 | *B. diazoefficiens* | AB984090 | [LC331638](https://www.ncbi.nlm.nih.gov/nuccore/LC331638) | ST3 |
| KG1-7 | *B. diazoefficiens* | AB984094 | [LC331639](https://www.ncbi.nlm.nih.gov/nuccore/LC331639) | ST3 |
| KM4-6 | *B. diazoefficiens* | AB984074 | [LC331640](https://www.ncbi.nlm.nih.gov/nuccore/LC331640) | ST3 |
| HK3-8 | *B. diazoefficiens* | AB983882 | [LC331641](https://www.ncbi.nlm.nih.gov/nuccore/LC331641) | ST4 |
| NK6 | *B. diazoefficiens* | AP014685 | AP014685 | ST5 |
| FK2-28 | *B. diazoefficiens* | AB984040 | [LC331642](https://www.ncbi.nlm.nih.gov/nuccore/LC331642) | ST5 |
| NG-3 | *B. diazoefficiens* | AB983991 | [LC331643](https://www.ncbi.nlm.nih.gov/nuccore/LC331643) | ST5 |
| YM2-8 | *B. diazoefficiens* | AB983977 | [LC331644](https://www.ncbi.nlm.nih.gov/nuccore/LC331644) | ST5 |
| YM2-30 | *B. diazoefficiens* | AB983979 | [LC331645](https://www.ncbi.nlm.nih.gov/nuccore/LC331645) | ST5 |
| KS1-5 | *B. diazoefficiens* | AB983947 | [LC331646](https://www.ncbi.nlm.nih.gov/nuccore/LC331646) | ST6 |
| FK1-7 | *B. diazoefficiens* | AB984028 | [LC331679](https://www.ncbi.nlm.nih.gov/nuccore/LC331679) | IS-inserted |
| MY1-5 | *B. diazoefficiens* | AB984081 | [LC331680](https://www.ncbi.nlm.nih.gov/nuccore/LC331680) | IS-inserted |
| TS2-3 | *B. diazoefficiens* | AB984002 | [LC331681](https://www.ncbi.nlm.nih.gov/nuccore/LC331681) | IS-inserted |
| YM2-1 | *B. diazoefficiens* | AB983971 | [LC331682](https://www.ncbi.nlm.nih.gov/nuccore/LC331682) | IS-inserted |
| USDA 123 | *B. japonicum* | AB830107 | AXVP01000508 | IS-inserted |
| USDA 124 | *B. japonicum* | AB100753 | KB893878 | IS-inserted |
| HK1-37 | *B. japonicum* | AB983870 | [LC331683](https://www.ncbi.nlm.nih.gov/nuccore/LC331683) | IS-inserted |
| USDA76^T^ | *B. elkanii* | KB900701 | - | - |

*^a^* NopP types are based on amino acid sequence patterns (see Supplementary Table 3). Strains of the “IS-inserted” type have insertion sequence (IS) elements located in the coding region of *nopP*.

**Supplementary Table 3. Differences in deduced NopP amino acid sequences among *Bradyrhizobium diazoefficiens* and *B. japonicum* strains.**

| Sequence type *^a^* | Amino acid residue *^b^* | | | | | | | | | | | | | | | | | |  | Number of strains *^c^* | |  | Nod *^d^* |
| --- | --- | --- | --- | --- | --- | --- | --- | --- | --- | --- | --- | --- | --- | --- | --- | --- | --- | --- | --- | --- | --- | --- | --- |
|  | 5 | 16 | 21 | 43 | 50 | 51 | 55 | 59 | **60** | **67** | 73 | 105 | 119 | 140 | 144 | **173** | 181 | **271** |  | *B. diazoefficiens* (22) | *B. japonicum* (33) |  |  |
| 110 | V | N | P | T | S | G | V | Q | L | Q | D | T | Q | T | K | Y | D | L |  | 4 | 5 |  | + |
| 122 | V | N | P | T | S | G | V | Q | R | R | D | T | Q | T | K | H | D | F |  | 4 | 4 |  | – |
| ST1 | V | N | P | T | S | G | V | Q | R | R | D | T | Q | T | K | H | D | L |  | 0 | 2 |  | – |
| ST2 | V | N | P | T | S | G | V | Q | R | Q | D | T | Q | T | K | Y | D | L |  | 0 | 19 |  | + |
| ST3 | V | N | P | S | S | G | V | Q | R | L | D | T | R | T | K | H | D | I |  | 3 | 0 |  | + |
| ST4 | V | N | P | T | S | G | V | E | R | Q | D | T | Q | T | K | Y | D | I |  | 1 | 0 |  | + |
| ST5 | I | S | S | T | A | R | D | E | R | Q | G | T | Q | A | R | H | H | L |  | 5 | 0 |  | + |
| ST6 | I | S | S | T | A | R | D | E | R | Q | G | S | Q | A | R | H | H | L |  | 1 | 0 |  | + |
| IS-inserted *^e^* | – | – | – | – | – | – | – | – | – | – | – | – | – | – | – | – | – | – |  | 4 | 3 |  | + |

*^a^* Nucleotide sequences of the *nopP* coding region were obtained from the NCBI GenBank database and the Japanese *Bradyrhizobium* culture collection^5^. Deduced amino acid sequences were aligned and the types were assigned on the basis of the sequence pattern. Strains of the “IS-inserted” type have insertion sequence (IS) elements located in the coding region of *nopP*.

*^b^* Numbers indicate positions in the NopP amino acid sequence (277 aa). Residues differing between USDA 110^T^ and 122 sequences are shown in bold. Residues shaded in gray differ from those of USDA 110^T^.

*^c^* Numbers in parentheses indicate the total number of tested strains.

*^d^* Nodulation phenotype of a representative strain(s) on *Glycine max* cv. Hardee (Supplementary Fig. 11).

*^e^* Homologous region of *nopP* was present in the genome but the CDS was not annotated due to insertion of an IS element.

**Supplementary Table 4**. Natural mutations in *nopP* in the genome of the *Bradyrhizobium diazoefficiens* Is-1 Tn5 mutants.

| Strain | Characteristic | *nopP ^a^* | Nucleotide change *^c^* | Amino acid change |
| --- | --- | --- | --- | --- |
| Is-1 | Wild-type strain | + | Not detected |  |
| 1C1 | Tn5 mutant | + | Not detected | – |
| 1C2 | Tn5 mutant | + | Not detected | – |
| 5C1 | Tn5 mutant | + | Not detected | – |
| 6C1 | Tn5 mutant | + | Not detected | – |
| 7C1 | Tn5 mutant | – |  |  |
| 7C2 | Tn5 mutant | + *^b^* | c.720insISBdi2 | – |
| 10C1 | Tn5 mutant | + | c.252C>A | p.Y84Ter |
| 10C2 | Tn5 mutant | + | Not detected | – |

*^a^* The presence (+) or absence (–) of the *nopP* coding region detected by PCR with the oligonucleotide primers nopP_F1 and nopP_R1 (Supplementary Data 2).

*^b^* The detected fragment was longer than expected.

*^c^* The sequences of PCR products were determined by Sanger sequencing with the oligonucleotide primers nopP_F1, R1, F2, and R3 (Supplementary Data 2), and compared with that of Is-1 (wild-type) in GenBank (accession number: LGUJ01000050).

**Supplementary Table 5.** Results of BLAST homology search of Rj2 protein.

| Species | Gene | Score | Query  cover | E value | Identity | Accession |
| --- | --- | --- | --- | --- | --- | --- |
| *Vigna radiata* var. *radiata* | LOC106757128 | 1466 | 99% | 0 | 71% | XM_022784298.1 |
| *Cajanus cajan* | LOC109818075 | 1436 | 99% | 0 | 69% | XM_020383471.1 |
| *Vigna angularis* | LOC108345610 | 1412 | 99% | 0 | 68% | XM_017584233.1 |
| *Phaseolus vulgaris* | PHAVU_004G028900g | 1394 | 99% | 0 | 67% | XM_007151169.1 |
| *Arachis ipaensis* | LOC107645897 | 1202 | 98% | 0 | 60% | XM_016350035.2 |
| *Arachis duranensis* | LOC107492123 | 1191 | 98% | 0 | 60% | XM_016113101.2 |
| *Medicago truncatula* | MTR_8g075440 | 1174 | 98% | 0 | 60% | XM_003629243.2 |
| *Cicer arietinum* | LOC101502635 | 962 | 97% | 0 | 50% | XM_004515171.2 |

TBLASTN search was conducted using amino acid sequence of Rj2 protein (GenBank accession no. ADF78112) as a query. GenBank database excluded the genus of *Glycine* was used. Only genes with the highest homology from one species were described in the list. Matches with percent identity >50% and query coverage > 95% were retained.

**Supplementary References**

1 Ohtsubo, Y., Ikeda-Ohtsubo, W., Nagata, Y. & Tsuda, M. GenomeMatcher: a graphical user interface for DNA sequence comparison. *BMC Bioinformatics* **9**, 376 (2008).

2 Tsukui, T. *et al.* The type III Secretion System of *Bradyrhizobium japonicum* USDA122 mediates symbiotic incompatibility with *Rj2* soybean plants. *Appl Environ Microbiol* **79**, 1048-1051 (2013).

3 Ishizuka, J., Yokoyama, A. & Suemasu, Y. Relationship between serotypes of *Bradyrhizobium japonicum* and their compatibility with *Rj*-cultivars for nodulation. *Soil Science and Plant Nutrition* **37**, 23-30 (1991).

4 Tsurumaru, H., Yamakawa, T., Tanaka, M. & Sakai, M. Tn5 mutants of *Bradyrhizobium japonicum* Is-1 with altered compatibility with *Rj2*-soybean cultivars. *Soil Science and Plant Nutrition* **54**, 197-203 (2008).

5 Shiina, Y. *et al.* Relationship between soil type and N_2_O reductase genotype (*nosZ*) of indigenous soybean bradyrhizobia: *nosZ*-minus populations are dominant in Andosols. *Microbes Environ* **29**, 420-426 (2014).

6 Kanehara, K. & Minamisawa, K. Complete genome sequence of *Bradyrhizobium japonicum* J5, isolated from a soybean nodule in Hokkaido, Japan. *Genome Announc* **5**, doi:10.1128/genomeA.01619-16 (2017).

7 Savka, M. A., Ravillion, B., Noel, G. R. & Farrand, S. K. Induction of hairy roots on cultivated soybean genotypes and their use to propagate the soybean cyst nematode. *Phytopathology* **80**, 503-508 (1990).

8 Schäfer, A. *et al.* Small mobilizable multi-purpose cloning vectors derived from the *Escherichia coli* plasmids pK18 and pK19: selection of defined deletions in the chromosome of *Corynebacterium glutamicum*. *Gene* **145**, 69-73 (1994).

9 Maekawa, T. *et al.* Polyubiquitin promoter-based binary vectors for overexpression and gene silencing in *Lotus japonicus*. *Mol Plant Microbe Interact* **21**, 375-382 (2008).

10 Figurski, D. H. & Helinski, D. R. Replication of an origin-containing derivative of plasmid RK2 dependent on a plasmid function provided in *trans*. *Proc Natl Acad Sci U S A* **76**, 1648-1652 (1979).

11 Wilson, K. J. *et al.* Glucuronidase (GUS) transposons for ecological and genetic studies of rhizobia and other Gram-negative bacteria. Microbiology 141, 1691–1705 (1995).

12 Kinoshita, E. Kinoshita-Kikuta, E. Takiyama, K. & Koike, T. Phosphate-binding tag: A new tool to visualize phosphorylated proteins. *Molecular & Cellular Proteomics*, **5**, 749-757 (2006).
