# Supplementary material for: Variation in bradyrhizobial NopP effector determines symbiotic incompatibility with Rj2-soybeans via effector-triggered immunity
Source: Nat Commun. 2018 Aug 7;9:3139. doi: 10.1038/s41467-018-05663-x (PMC6081438; doi:10.1038/s41467-018-05663-x)
Supplement: Supplementary file 3 — Description of Additional Supplementary Files [file 41467_2018_5663_MOESM3_ESM.pdf]

## Description of Additional Supplementary Files

File Name: Supplementary Data 1
Description: Bacterial strains and plasmids used in this study.

File Name: Supplementary Data 2
Description: Oligonucleotide primers used in this study.
